# Ionomer Interphase Layers Enable Efficient Anion-Exchange Membrane Water Electrolyzer Operation at Low pH

**DOI:** 10.1021/acs.energyfuels.5c00396

**Published:** 2025-04-23

**Authors:** Arthur P. L. Thévenot, Thilo Reiter, Trung Ngo Thanh, Lisa Titze, Cristina Cazzaniga, Fabio Dionigi, Peter Strasser

**Affiliations:** † Department of Chemistry, Chemical Engineering Department, Technical University Berlin, 10623 Berlin, Germany; ‡ Industrie De Nora S.p.A.,Via Bistolfi 35, 20134 Milan, Italy

## Abstract

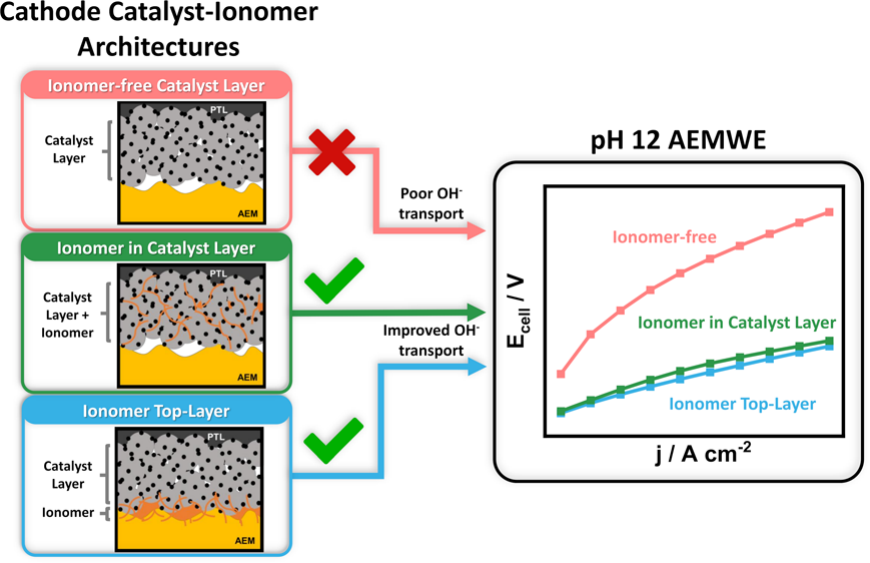

Anion-exchange membrane water electrolysis (AEMWE) is an emerging green hydrogen technology. As of today, AEM water electrolyzers operate using highly alkaline electrolytes. Design strategies to operate AEMWE systems sustainably under lower alkalinity toward pure water conditions have become a scientific priority. Under low-alkaline conditions, the alkaline-exchange ionomer (AEI) is, in addition to the AEM, the key ion-transport medium inside the AEMWE cell. While prior work addressed ion transport and the ionomer-catalyst interface at the anode in low-pH AEMWEs, a thorough investigation at the cathode side, including different AEI architectures, received limited attention. In this contribution, we explore the impact of AEI architectures in AEMWE cathodes using an ionomer and a membrane that are both commercially available. We demonstrate separate ionomer top layer (ITL) interphases placed between the cathode catalyst layer and the membrane as the most effective strategy toward high cell performance under low pH feeding. ITLs enabled performance benefits even at pH 14, which leads us to perceive their mechanistic role as an ion-transport buffer enabling ready ion migration from the cathode to the anode. Our insights on the ITL architecture will aid the design of AEMWE cells for sustained efficient operation under pure water feeds.

## 1 Introduction

Demand for low-carbon, in particular green hydrogen, is expected to grow significantly to meet the challenges of reaching a net-zero emission (NZE) energy system, in which fossil fuels will be replaced with molecular hydrogen or its carbonaceous derivatives.^1^

Among green hydrogen production technologies, the anion-exchange membrane water electrolyzer (AEMWE) combines the benefits of proton-exchange membrane water electrolyzer and alkaline water electrolyzer (PEMWE and AWE, respectively), such as (i) a solid polymer electrolyte membrane enabling high efficiency (70–80%) and H_2_ purity (>99.9%) and (ii) the use of nonprecious catalyst materials.^2^ Indeed, to date, advanced AEMWEs have demonstrated high current densities (>5 A cm^–2^),^3^ low ohmic resistances, and exclusive usage of sustainable materials,^4^ resulting in an overall reduced carbon footprint. However, high-performance AEMWEs continue to suffer from lower ionic conductivity, compared to PEMWEs, that contributes to their lower performance in comparison to PEMWEs. In addition, AEMWEs continue to require liquid alkaline electrolyte for stability, in contrast with PEMWEs that operate with purified water and pose challenges in the corrosion resistance of the system components. This is why the design and stable operation of AEMWEs under lower-pH liquid electrolyte conditions represent a major research priority.^2−6^

Even though liquid KOH concentrations in today’s AEMWEs are significantly lower than those employed in AWEs, they typically still range around 1 M KOH to ensure proper functionality of the anode catalyst, to reduce the ohmic losses across the membrane, and to optimize the ion transport across the catalyst layer (CL)-electrolyte membrane interface.^7^ This corrosive electrolyte can rapidly degrade catalysts, membrane, and ionomer but also reduces the lifetime of other cell components resulting in leakage risks and higher maintenance costs. Such challenges can be overcome by feeding the cell with low alkaline (low pH) or neutral pure water as electrolytes.^5,7−9^ Switching from corrosive alkaline conditions to pure water feeding comes with its own challenges, such as increased overpotentials due to reduced ion conductivity combined with catalyst leaching/structural reconstruction due to metal leaching.^6,7,10−13^ To date, solutions to tackle the electrolyte challenge largely involved use of lower-pH KOH or K_2_CO_3_ (<10 wt %) electrolytes being a compromise for performance stability.^7,9−12^

Recently, the hydroxide ion-transport characteristics between the anode CL and the membrane, more specifically the effective interfacial area of contact where hydroxide ion transport occurs, has received major attention in attempts to clarify the origin of the alkalinity requirements. Efforts to address the interface between the anode catalyst and AEM using a number of different anion-exchange ionomers (AEIs) incorporated into the CL were reported by Motz et al., who studied the influence of the chemical structure and physical properties of polymer electrolytes on the performance and durability of AEMWEs under 1 wt % K_2_CO_3_/1 wt % KOH and DI water.^14^ The authors mentioned the ionomer-catalyst interactions as a key factor for activity and stability in low-alkaline/pure water feed AEMWEs and showed that AEIs with the least catalyst–ionomer interaction are the most beneficial for AEMWE’s performance. Using a customized ionomer top-layer (ITL) on a substrate-grown anode, Wan et al. highlighted the role of AEIs as “transport highways” to boost transport of liquid/gas, hydroxide ions, and electron in the electrode, resulting in a high current density of 1900 mA cm^–2^ at 1.90 V in a pure water-fed AEMWE.^15^ They reported that the role of AEI is to increase the local OH^–^ concentration on the integrated electrodes’ surface and facilitate the oxygen evolution reaction (OER) for pure water-fed AEMWEs. Optimizing the anode for pure water AEM electrolysis, Xu et al. highlighted the importance of ionomer distribution in the anode catalyst ink using ionomer in the CL as well as an ITL.^12^ Lindquist et al. studied anodic AEI degradation under different CL additives and electrolyte feed and identified ionomer oxidation as the dominant degradation mechanism for all hydroxide exchange membrane-based devices operating in nominally pure water.^9^ An ITL was sprayed on the ionomer-containing CLs on both sides, and all hydrocarbon-based AEIs oxidized rapidly, losing both backbone and cationic side-chain groups. The high rate of degradation can be in part attributed to the combination of the strongly oxidizing environment and high pH. They finally showed that oxidation-resistant CL binders, such as PTFE, improved cell stability but also reduced the voltage efficiency due to the resulting high ion-transport resistance. In all, while AEIs inside and on top of the anode CL have been widely recognized as a viable advance toward low-pH AEMWE operation, there is a lack of insight and understanding of the role and impact of AEIs in and on top of the cathode of AEMWEs. Moreover, ion transport at the cathode is emerging as the actual bottleneck in low-pH AEMWEs and requires innovative solutions. Zheng et al. operated an AEMWE for over 550 h at 1 A cm^–2^ with a cell voltage of 1.82 V under pure water feed using PGM-free electrocatalysts and an ionomer layer above the cathode CL.^16^ In 1 M KOH conditions, Lou et al. investigated the influence of an additional ITL in combination with ionomer-containing CLs in CCS, CCM, and CCS-CCM configurations. An ionomer layer on either the cathode or anode CL was found to improve the performance in CCM but led to a performance loss in CCS configuration.^17^

In this work, in contrast to previous studies focusing on one specific cathodic ionomer architecture, we explore the role and quantify the impact of two distinct types of AEI layers on the cathode, ionomer incorporated in the catalyst layer (ICL) and ITL, on the performance and durability of low-pH operated AEMWEs using state-of-the-art benchmark components.^18−25^ We investigate the effects of AEI loading and the location of ICLs and ITLs on the AEMWE cathode, identifying optimized cell design parameters for low-pH operation conditions. Finally, we reveal the influence of cathodic ITL on the performance improvement by comparing cell activity under low- and high-pH feeds.

## 2 Experimental Section

### 2.1 Materials

For the NiFe-LDH synthesis, DMF (HPCL grade, 99.9 + %) was purchased from VWR, absolute ethanol (99.9 + %) was purchased from Chemsolute, Fe(NO_3_)_3_·9H_2_O (99 + %) was purchased from Acros Organics, and Ni(OAc)_2_·4H_2_O (99%) was purchased from Chempur. The Ni felt as well as the Sustainion XA-9 ionomer solution and X37–50T AEM were obtained from Dioxide Materials. KOH pellets (99.98% metal basis) used for electrolyte preparation of electrochemical single-cell tests were obtained from Thermo Fisher Scientific.

### 2.2 Anode Preparation

#### 2.2.1 NiFe-LDH Synthesis

NiFe-LDH OER electrocatalyst powders were prepared through a modified solvothermal route reported previously by Dresp et al.^26^ Briefly, a mixture containing DMF (208.0 mL), Ni(OAc)_2_·4H_2_O (62.5 mL, 0.6 M), and Fe(NO_3_)_3_·9H_2_O (12.5 mL, 0.6 M) was stirred for 1 h. Afterward, ultrapure water (Milli-Q-H_2_O, 278.0 mL) and DMF (139.0 mL) were added to the mixture and stirred for an additional 15 min. The obtained dark red-brown solution was transferred into a Teflon vessel for hydrothermal treatment in a microwave (Anton Paar; 1 h at 120 °C and then 1 h at 160 °C). The product was centrifuged and subsequently washed with ethanol and Milli-Q-H_2_O. After freeze-drying, the product was obtained as a yellow powder.

#### 2.2.2 Porous Transport Layer (PTL) Cutting and Cleaning

Ni fiber felts were laser cut into 5 cm^2^ square-shaped PTLs and cleaned using an ultrasonic bath, applying 5 min sonication steps in the following solutions subsequently: 1:1 vol. mixture of *i*PrOH/H_2_O, Milli-Q-H_2_O, 3 M HCl solution, and again Milli-Q-H_2_O. Cleaned Ni fiber felts were dried in an oven at 60 °C and stored in a N_2_ box.

#### 2.2.3 CL Coating

##### 2.2.3.1 Catalyst Ink Preparation

NiFe-LDH (40.0 mg) was dissolved in 3 mL of EtOH. Sustainion XA-9 ionomer solution (90.0 mg of 5 wt % solution in EtOH) was added to yield the final 10 wt % (*w*_ionomer_/*w*_total solid content=catalyst+ionomer_) ink. The resulting ink was then tip-sonicated (Branson SFX250 Digital Sonifier) at room temperature for 20 min at 20% power.

##### 2.2.3.2 Catalyst Ink Spraying

Cleaned Ni fiber felt was taped on a 5 cm^2^ square-shaped mask and supported magnetically on a heating plate. The catalyst ink was spray-coated on the PTL using an airbrush under N_2_ gas flow, and the heating plate’s temperature controller (CNL) was set to 80 °C. A catalyst loading of 2.5 ± 0.2 mg cm^–2^ was calculated from the mass difference in the dry state. The mass of the anode prior to spraying was measured after heating the PTL at 80 °C for 10–15 min and then cooling down at room temperature (RT) for 3–5 min to remove traces of moist.

#### 2.2.4 ITL Coating

In 3 mL of EtOH was diluted a Sustainion XA-9 ionomer solution (90 to 360 mg of 5 wt % solution, depending on the desired ITL loading on the PTL). The ITL ink was sprayed over CL as described above for the CL coating.

### 2.3 Cathode Preparation

The cathodes used for this series of experiments were the industrially produced cathode by De Nora (ionomer-free) and the same cathode with the CL modified by the presence of Sustanion XA-9 ionomer. The commercial cathode consists of a carbon cloth substrate, a carbon-based microporous layer, and a reactive layer containing Pt/C as the catalyst.

For the modification of the reactive layer, an aqueous ink containing Pt/C and an ionomer solution (Sustanion XA-9) in various percentages (5, 10, and 20%) calculated based on the Pt content was used. This ink was then deposited on the same substrate with a microporous layer as the one used in the industrial cathode. The platinum content in the CL is 0.4 ± 0.02 mg cm^–2^ for all samples, calculated as the difference in dry weight.

The ITL was sprayed on the CL as mentioned above, with the ionomer mass loading in the EtOH solution varying from 45 to 395 mg of 5 wt % solution.

### 2.4 Membrane Electrode Assembly (MEA) and Electrolyzer Operation

An in-house benchtop setup was used, which features a single electrolyzer cell (Hydron Energy) composed of titanium end plates and titanium current collector plates (5 cm^2^ active area; parallel flow-fields), with 0.5 mm Black Ice Cube Sealing gaskets between end plates and collector plates. To condition the membrane, Sustainion X37–50T AEM was soaked in 1 M KOH for 20 h, replacing the solution with fresh 1 M KOH solution 50 min prior to assembly in the electrolyzer single cell. The ion-exchanged membrane was sandwiched between the anode and the cathode under a torque value of 12.5 N m. Blue Ice Cube Sealing gaskets were used between the membrane and the anode (0.5 mm gasket)/cathode (0.35 mm gasket) flow-fields. Detailed cell assembly procedure is described in Figure S10. A constant cell temperature of 60 °C was set by heating cartridges, mounted on both end plates of the cell, and a temperature controller (CNL). The electrolyte flow was adjusted using two Simdos 10 diaphragm metering pumps (KNF) at a flow rate of 50 mL min^–1^. The electrochemical testing was conducted using a Reference 3000 potentiostat (Gamry) equipped with a high-current 30k Booster. Figures S13 and S14 describe, respectively, the used activity and stability electrochemical testing protocols. Each reported chronopotentiometry (CP) value point from the polarization curve was calculated by averaging the corresponding 10 s CP step of three consecutive loops, and all potentiostatic electrochemical impedance spectra (PEIS) measured during these loops were used to calculate the respective high-frequency resistance (HFR) values. Regarding durability tests, the HFR values were obtained from the PEIS measurement after each 20 h CP.

### 2.5 SEM Characterization of “Post-Mortem” MEAs and “As-Prepared” Electrodes

The morphology of the tested MEAs (“post mortem”) and untested samples (“as-prepared” anodes and cathodes) was examined using scanning electron microscopy (SEM) with an SEM/FEG Inspect F50 FEI microscope. Each sample was characterized at different magnifications, focusing on the cross-section of the CL to better understand the possible modifications in the reactive layer structure after testing under various conditions.

## 3 Results and Discussion

### 3.1 Benchmark Tests under Low-pH Conditions

**Figure 1:**
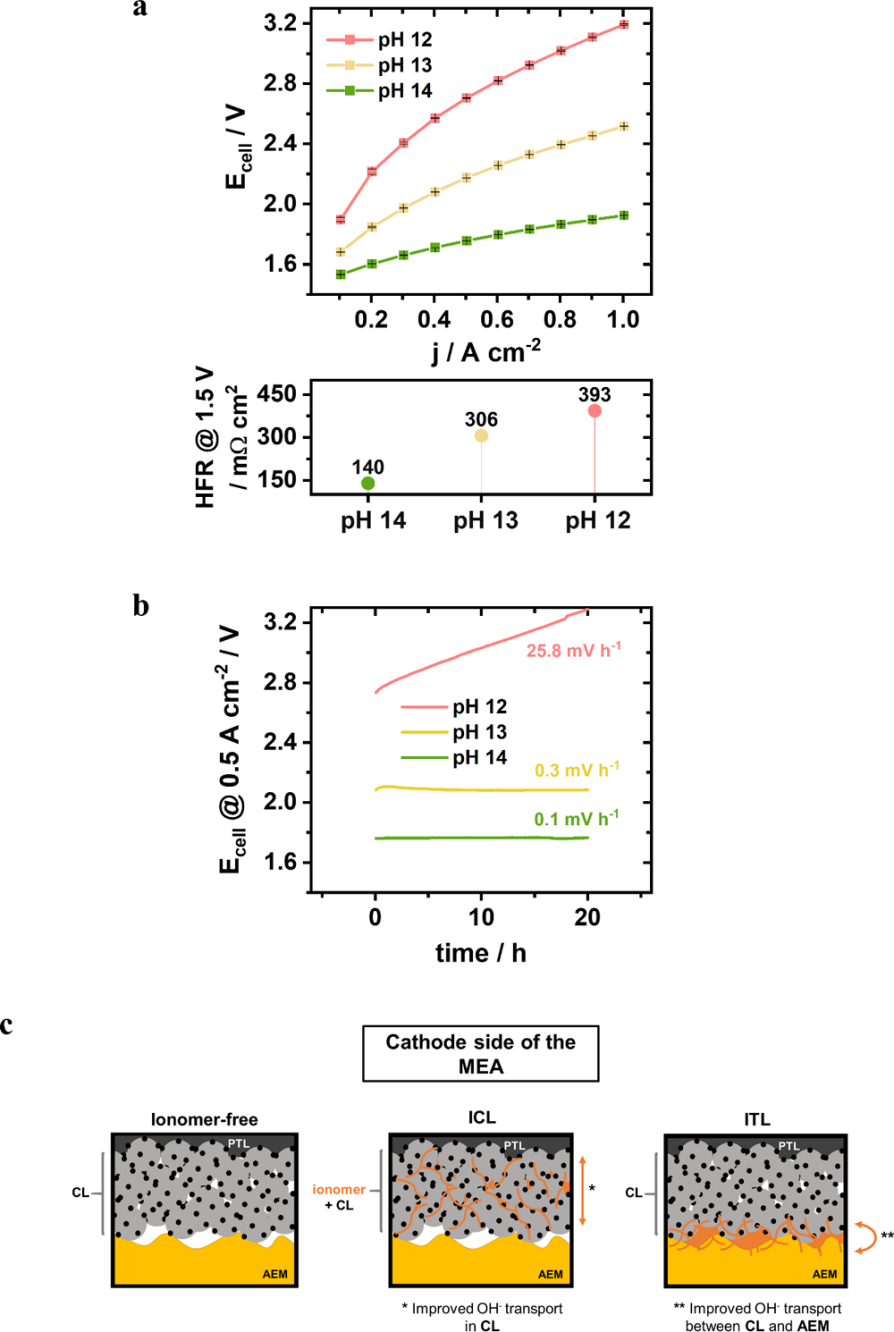
Single-cell performance of the MEA using binder-free Pt/C (∼0.4 mg_Pt_ cm^–2^) on carbon cloth as the cathode, Sustainion X37–50T as the membrane, and NiFe-LDH (∼2.5 mg cm^–2^) with 10 wt % Sustainion XA-9 ionomer (wt % relative to the total solid content in the ink) on Ni felt as the anode, tested in different electrolyte feeds varying from pH 14 (1 M KOH, green) to pH 13 (0.1 M KOH, yellow) and pH 12 (0.01 M KOH, red) at 60 °C and symmetric feed mode with 50 mL min^–1^ flow. (a) Polarization curve from 0.1 to 1 A cm^–2^ with 0.1 A cm^–2^ CP steps, calculated by taking the mean value of three consecutive polarization curves with the black error bars representing the standard deviation. The respective HFR values obtained from PEIS measurements @ 1.5 V are shown below. (b) Durability study of a 20 h current hold @ 0.5 A cm^–2^ under the three different pH values of the feed. The shown degradation rates are calculated considering the last 10 h of every step. (c) Simplified scheme for visualization of the three cathodes’ design investigated in this study: the ionomer-free cathode (left), the ionomer-incorporated cathode catalyst layer (ICL, middle), and the ionomer top-layer on the cathode catalyst layer (ITL, right).

Our benchmark cell design incorporated a commercial ionomer-free carbon cloth-supported Pt/C cathode layer supplied by De Nora, a Sustainion X37–50T AEM, and a Ni felt-supported and spray-deposited NiFe-LDH anode with the AEI binder (10 wt % (*w*_ionomer_/*w*_catalyst+ionomer_) Sustainion XA-9). The benchmark AEMWE cell was measured using three distinct pH feeds: 1 M KOH (pH 14), 0.1 M KOH (pH 13), and 0.01 M KOH (pH 12). The catalysts for the cathode and the anode are the most active electrocatalysts for the hydrogen evolution reaction and OER, respectively, in alkaline electrolytes,^18−23^ while X37–50T constitutes a well-studied and commercially available AEMWE.^7,24,27,28^Figure 1a depicts the AEMWE cell polarization curves at the three distinct pH conditions, whereas Figure 1b shows the respective durability tests during a 20 h current hold @ 0.5 A cm^–2^ with the degradation rates calculated from the last 10 h.

While the polarization curve at pH 14 remained below 1.9 V @ 1 A cm^–2^, the drop in OH^–^ concentration in the feed by 1 order of magnitude led to an increased ohmic resistance and a significant activity loss. Further feed dilution to pH 12 caused cell voltages over 3 V at current densities above 0.8 A cm^–2^, which is a sharp and intolerable drop in the AEMWE cell performance.^10^ Regarding the durability tests in Figure 1b, a moderate degradation rate of 0.1 mV h^–1^ is observed after 20 h in pH 14, but shifting to pH 13 results in a 3 times higher degradation rate and a more pronounced equilibration/break-in phase at the beginning of the measurement. At pH 12, the degradation of the system drastically accelerates further with a degradation rate of 25.8 mV h^–1^, resulting in a cell voltage above 3 V @ 0.5 A cm^–2^ after less than 9 h and reaching 3.29 V after 20 h.

### 3.2 Cathode ICL and ITL Low-pH Performance Hypothesis

**Figure 2:**
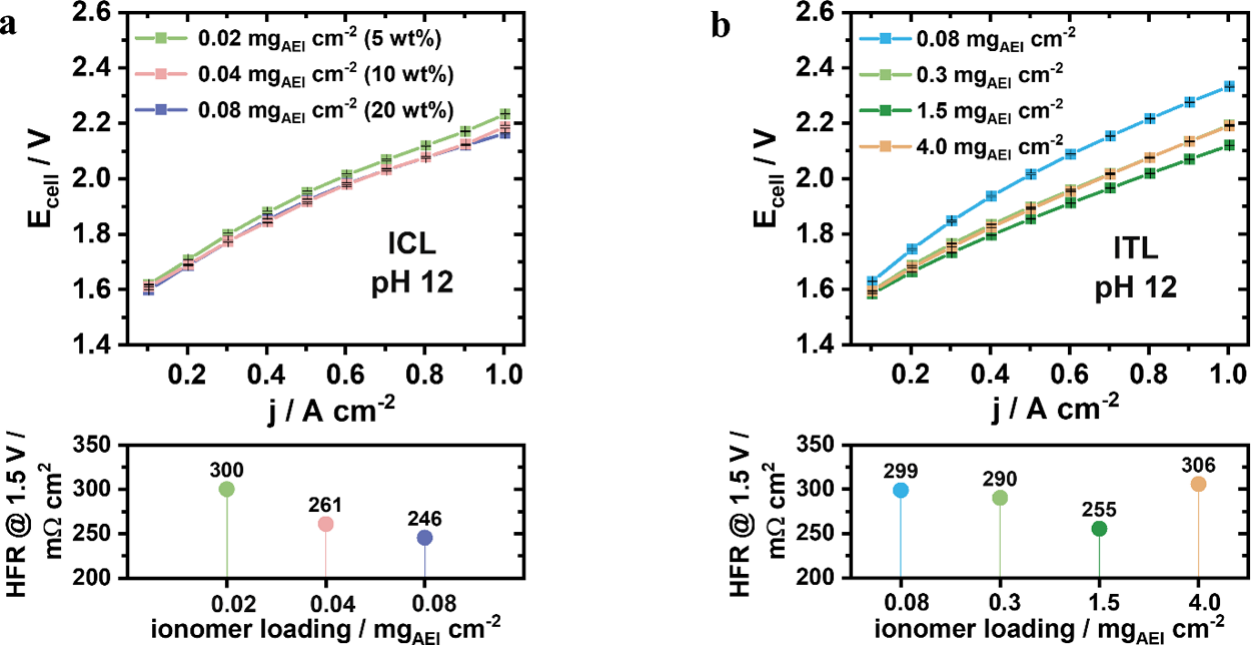
Study in pH 12 of the two ionomer approaches at the Pt/C cathode (∼0.4 mg_Pt_ cm^–2^) with (a) ICL with the following ionomer loadings: 0.02 mg_AEI_ cm^–2^(=5 wt %, green), 0.04 mg_AEI_ cm^–2^(=10 wt %, light red), and 0.08 mg_AEI_ cm^–2^(=20 wt %, violet) and (b) ITL over the binder-free Pt/C electrode with the following loadings: 0.08 mg_AEI_ cm^–2^(light blue), 0.3 mg_AEI_ cm^–2^(dark blue),1.5 mg_AEI_ cm^–2^(green), and 4.0 mg_AEI_ cm^–2^(yellow). The respective HFR values measured @ 1.5 V are shown below, and the stated weight percentages are with respect to the Pt content in the CL.

While numerous earlier studies attributed low-pH AEMWE degradation to anode issues, we hypothesize that the hydroxide ion transport from the cathode CL across the membrane to the anode is actually the limiting factor in cell performance.^15,16^ Following this idea, we explored how the deployment of various amounts of alkaline exchange ionomer (AEI) at the cathode affects the transport of hydroxide ion from the CL into the membrane. Three different design strategies were used to deploy AEI in and on the cathode CL: (i) no incorporation of AEI binder in the cathode, (ii) incorporating the AEI inside the cathode catalyst layer (ICL), and (iii) deploying AEI in form of a separate interphase top-layer (ITL) between the CL and the membrane, as illustrated in Figure 1c. Figures S3–8 show the SEM images of all three configurations, both before performance testing and after durability measurement, that are discussed in Supplementary Discussion 1. The ICL approach is a well-established practice using powder catalysts, wherein the role of the ionomer is primarily to act as a binder enabling ion transport across the CL. By contrast, the ITL interphase strategy is a recent novel approach in AEM water electrolysis.^9,12,15,16^ In the case of high pH, very conductive liquid electrolyte feeds, thick ITL designs can be regarded as an extension of the membrane, thus prolonging the pathway of ion transport causing added ohmic resistance. Thin ITLs, in contrast, should show benefits for low-pH AEMWE liquid feeds. The results of testing this hypothesis are shown in Figure 2. Table S2 shows the conductivity of the studied electrolyte solutions and of the Sustainion X37 membrane.

### 3.3 ICL Performance

The incorporation of small ionomer loadings (0.02 mg_AEI_ cm^–2^ = 5 wt %) into the cathode’s Pt/C CL, i.e., the ICL approach (Figure 2a), led to a significant performance improvement of the AEMWE cell, reflected by a voltage drop by about 1 V @ 1 A cm^–2^ along with a lower ohmic HFR value compared to the ionomer-free cathode benchmark cathode. Increasing the amount of ionomer in the CL to 0.04 mg_AEI_ cm^–2^ (=10 wt %) results in further cell improvement with the optimal AEI loading ranging around 0.08 mg_AEI_ cm^–2^ (=20 wt %). The observed performance boost suggests that the ionomer inside the CL is able to offset mass transport limitations at the cathode induced by the low-pH electrolyte. During AEMWE operation, the ionomer acts as an ion pathway, facilitating the transport of generated hydroxide ions from the cathode to the membrane.^12,15^

### 3.4 ITL Performance

The ITL approach can be regarded as a distinct AEI interphase between the CL and the AEM. A thin ionomer interphase layer (∼0.08 mg_AEI_ cm^–2^) was able to induce a quite similar activity enhancement as the ICL approach under optimized conditions (blue curve in Figure 2b). This surprising result reveals that the hydroxide mass transport in the bulk of the CL is not the sole performance-limiting kinetic factor, but the poorly understood interface between the membrane and the cathode CL also plays a critical role.

ITL interphases yielded lower ohmic cell resistances, with cell performance peaking at 1.5 mg_AEI_ cm^–2^ (Figure 2b, *dark green*). As thicker ITL can be interpreted as membrane extension, further thickening of the ITL interphases increases cell resistance and leads to a deterioration of activity (Figure 2b, *yellow*).

Interestingly, the ITL and ICL approaches show very different demands of ionomer as well as layer morphology (for more details, see Supplementary Discussion 1), and further experiments are needed to conclusively explain the performance difference between the ITL and ICL. The different demand of ionomer emphasized the contrasting behavior of both ionomer architectures.

### 3.5 ITL Approaches at the Anode

To check the impact of our ITL approach on the anode side of our benchmark AEMWE design, we added an ITL interphase on top of the AEI-incorporated NiFe-LDH CL (that is, in between the anode CL and membrane) (Figure S1). This approach showed mainly detrimental performance effects, indicating that the AEI deployed inside of the anode CL was fully sufficient to ensure hydroxide transport from the membrane to the catalyst. Merely using 1.5 mg_AEI_ cm^–2^ ITL, a slight improvement over the benchmark design was observed. Finally, deploying an ITL interphase on both the anode and cathode did not further improve the AEMWE performance (Figure S2).

### 3.6 Stability of ICL and ITL Interphases

**Figure 3:**
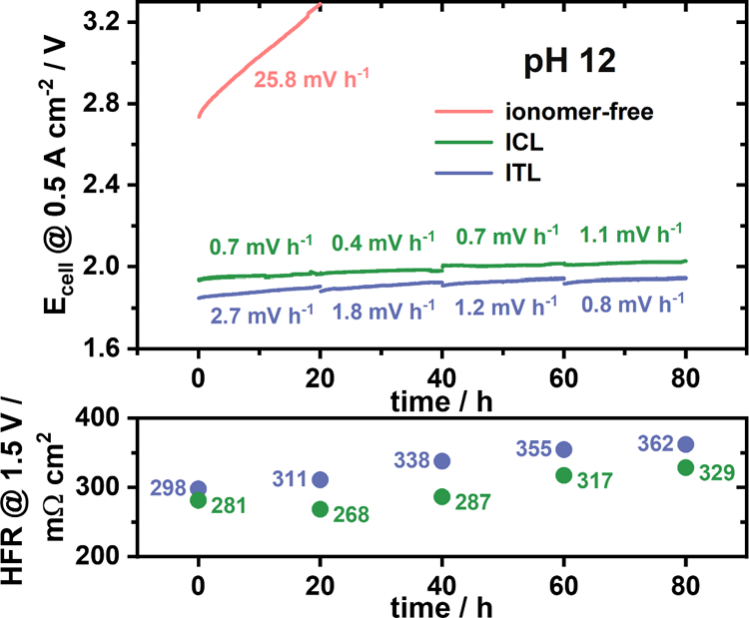
Comparison of the durability of the ICL and ITL designs at the cathode with the best ionomer loading in both approaches (ICL, 0.08 mg_AEI_ cm^–2^ = 20 wt %, green; ITL, 1.5 mg_AEI_ cm^–2^, violet) over 80 h at 60 °C. The current hold is divided into 4 × 20 h steps, and after each step PEIS @ 1.5 V are measured. The degradation rates are calculated considering the last 10 h of every measurement. As a reference, the stability of the ionomer-free cathode at pH 12 (red) is plotted.

To learn more about the durability of the cathodic ICL and ITL interphase layer designs, either approach underwent an 80 h AEMWE single-cell stability test at 0.5 A cm^–2^ (Figure 3, *green and blue traces*), along with the binder-free-cathode benchmark test (red trace). The current hold test was divided into 4 × 20 h steps with PEIS @ 1.5 V being recorded between each step to monitor the evolution of the cell HFR. Over the entire test duration, both approaches did not show any sign of sudden cell collapse observed for (ionomer-free-cathode) benchmark cells under the test conditions. Instead, the AEMWE cell revealed moderate degradation trajectories. The first 20 h of the ICL stability test resembled a break-in phase, followed by the lowest degradation rate (0.4 mV h^–1^) and lowest ohmic cell resistances from 20 to 40 h. Even though the degradation rates did not reveal a monotonic trend over 60 h, the final test segment was characterized by a larger degradation rate of 1.1 mV h^–1^. By contrast, the ITL interphase design set out first with higher degradation rates of 2.7 mV h^–1^ over 20 h but followed by favorable voltage degradation rates down to 0.8 mV h^–1^. The HFR values increased only slightly over the total test time, which could be the benefit from less oxidative ionomer degradation under lower alkaline conditions.^9^ It is remarkable that over the 80 h testing duration and despite having a higher HFR, the ITL remained superior in the overall cell performance. Further investigations need to be conducted addressing the different behavior.

### 3.7 Mechanistic Insights into the Role of ITL Interphases

**Figure 4:**
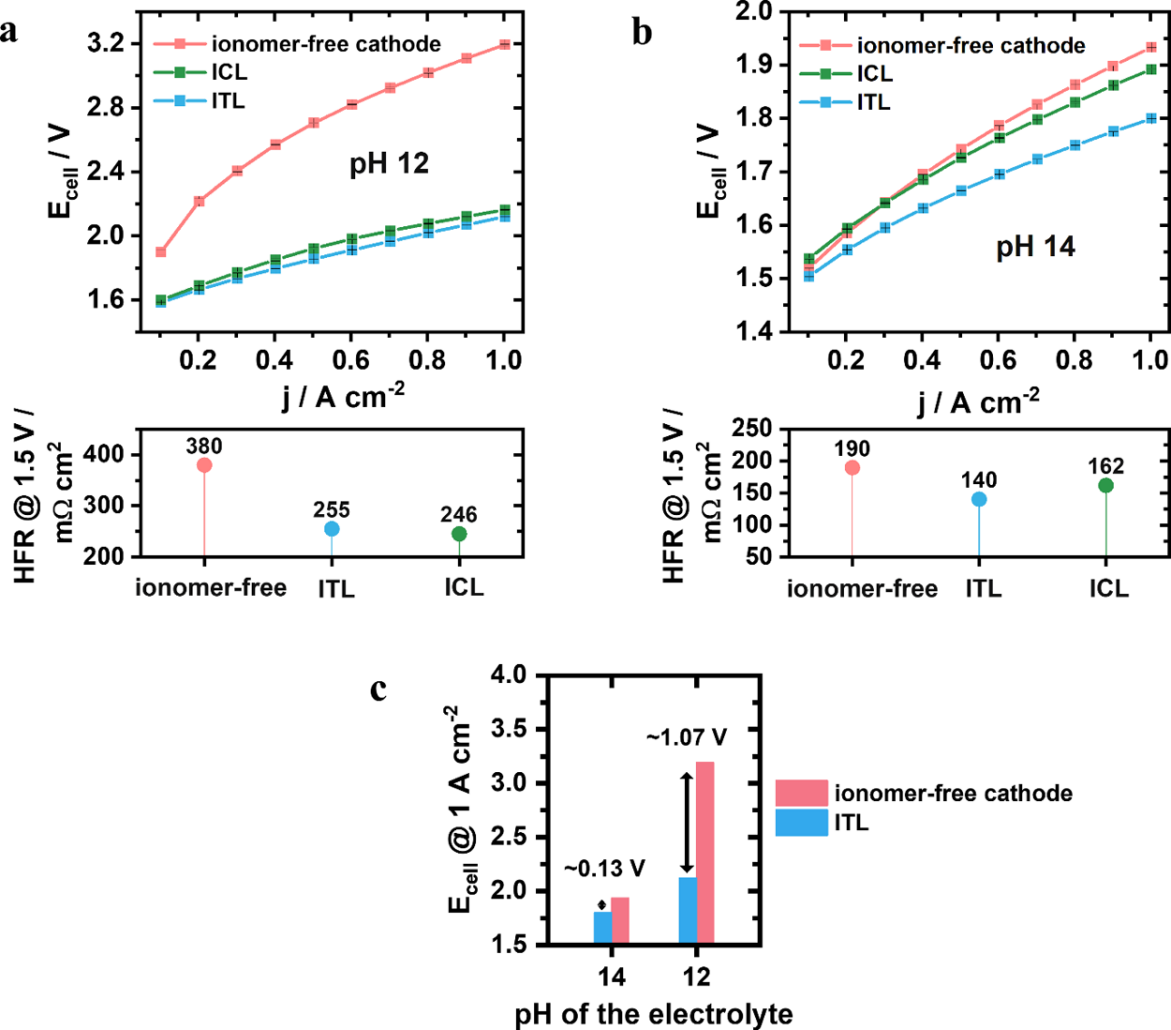
Influence of the ITL (1.5 mg_AEI_ cm^–2^, blue) compared with the ICL (0.08 mg_AEI_ cm^–2^, green) and the ionomer-free cathode (red) in terms of activity in (a) pH 12 and (b) pH 14 with the respective HFR measured @ 1.5 V. (c) Representation of the voltage drop @ 1 A cm^–2^ between the binder-free cathode and its ITL counterpart under high- and mild-alkaline feeds.

To deconvolute the functional role of ITL interphases better, we contrasted the cell performance of the optimized ICL and ITL designs under low- and high-pH conditions. Figure 4a highlights the superior activity of both the ICL and ITL cathodes over their binder-free counterpart, with the ITL being the best performing in the considered current density region. To correlate the observed behavior back with the well-established conditions at pH 14, both cathode designs were subsequently investigated in default 1 M KOH-electrolyte feeds (Figure 4b).

At low current densities, deployment of cathode ICL interphases resulted in slightly lower cell performance compared to the ionomer binder-free benchmark AEMWE cathode design. This suggests that, under pH 14 electrolyte conditions, the hydroxide ion transport from the cathode CL to the membrane in the ionomer-free benchmark design was optimal, whereas the added AEI interphase negatively impacted the cell voltage due to lower specific ionomer conductivity compared to that of a 1 M KOH solution (Table S2). Such observation correlates with the literature; Titheridge et al. report that the beneficial conductivity of 1 M KOH supporting electrolyte can be negated by the excess ionomer, impede the interfacial charge transfer, and may decrease the effective surface area of electrochemical reactions by encapsulating catalyst particles.^29^

At high current densities, on the other hand, the ICL design is superior to the ionomer-free design, resulting in better performance and a decrease in HFR. Such improvement can be due to the enhanced porosity of the CL from the ionomer incorporation that is observed by SEM in electrodes prepared with ICL design (Figure S3).

Unlike the ICL design, deploying the ITL interphase at pH 14 benefits cell performance across the entire voltage window and test time. This observation confirmed that even under highly conductive pH 14 electrolyte feeds, the additional AEI ITL interphase between the cathode CL and the membrane improves OH^–^ transport. This supports a mechanistic hypothesis that ITL interphases support the microscopic ion pathways between the CL and the membrane. We speculate that ITL interphases act as nonrigid polymeric interlayers, where uneven CL surfaces become smoothly linked to the membrane surface. It should be noted that in contrast to Lou et al., the ITL cathode discussed here has no additional ionomer in the CL.^17^ This reinforces our hypothesis that the ITL is especially beneficial in ionomer-scarce systems. Undoubtedly, the results emphasize the importance of the ionomer to form intimate contact between the CL and the membrane, also highlighted by Fortin et al.^30^

Figure 4c summaries the dramatic AEMWE cell voltage performance benefits over the ionomer binder-free benchmark originating from our ITL interphase design (Figure 1c) at pH 12 (>1 V) and pH 14 (∼0.13 V) electrolytes. The performance benefits are more pronounced at low-pH feeds, where liquid ion conductivities trail those of the fully humidified ITL (Table S2). The ITL interphase offers active electrode-sized contact areas to both CL and membrane direction. Thereby, the ITL succeeds in mediating enhanced OH^–^ transport.

## 4 Conclusions

This work addressed performance losses due to ion-transport challenges of AEMWE when operated using low-pH electrolyte feeds. Under these conditions and using our benchmark AEMWE, we identified the ionomer-free cathode as a key area for improvement in optimizing water electrolysis. The observed decrease in cell performance was addressed by investigating the effects of two distinct AEI/CL electrode architectures on both performance and durability, which were significantly improved. One AEI/CL design involved incorporating the AEI within the CL (ionomer-catalyst layer, ICL), while the other design approach added an ionomer interphase layer on top of the CL toward the membrane (ionomer top layer, ITL). The novelty of our work consists in (1) comparing both AEI architectures (ICL and ITL) on one same electrode (cathode) in a low-pH electrolyte and (2) in a relevant benchmark AEMWE. The first offers deeper insights into the understanding of the identified performance limiting factor, while the second approach using benchmark components (i.e., commercial membranes and ionomers) allows better comparative studies between different laboratories, potentially leading to a broader understanding of the investigated fundamental mechanisms.

First, we examined the AEMWE performance with an ionomer-free cathode under reduced alkalinity. The cell efficiency decreased drastically from 1.93 V at pH 14 to 3.19 V at pH 12, coupled with poor durability. We then confirmed that the mere presence of the ionomer in either ICL or ITL architecture, despite the possibility of site blocking, yielded a significant performance benefit under low-pH alkaline feeds over the ionomer-free benchmark that we attributed to an improved OH^–^ ion transport. This warranted a closer look at the spatial ionomer distribution at the cathode under hydroxide-deficient conditions.

We then compared the performance differences between the ICL and ITL electrode designs. The ITL showed an enhanced performance over the ICL under reduced alkalinity feeds. These data led us to conclude that hydroxide ion transport limitation is not primarily only an issue of the bulk CL but concerns, first and foremost, the interfacial space between the CL surface and the membrane. The beneficial impact of the ICL and ITL architectures was sustained over the entire 80 h of test time. Further investigations are required to fully understand the different properties of the ITL and ICL, such as layer thickness and porosity. The results clearly demonstrate that an ionomer-containing cathode is required under low-alkaline conditions and show that both ICL and ITL are valid approaches. The optimal choice of configuration is most likely dependent on the used ionomer, membrane, and catalyst material.

Finally, the AEMWE cell performance of both ICL and ITL designs was assessed at full pH 14 alkalinity in order to gain insights into the role of the AEI phase. Under such conditions, the interphase modification induced by the ITL revealed performance benefits over the test campaign, indicating that managing ion transport at the CL/membrane merits closer attention in the future. Our study should entice future work to make the cathode CL/membrane a priority in low-pH AEMWE development.

Based on our new insight on the importance of ion transport at the cathode CL/membrane interface, we are pursuing work on new ITL interphase architectures for pure water feed conditions.

## Supporting Information Available

Electrochemical study of ITL on the anode and on both electrodes; SEM characterization of “as-prepared” electrodes and “post-mortem” MEAs; additional information regarding materials and methods; and conductivity of electrolyte solutions and AEM (PDF)
