# Supplementary material for: Ionomer Interphase Layers Enable Efficient Anion-Exchange Membrane Water Electrolyzer Operation at Low pH
Source: Energy Fuels. 2025 Apr 23;39(17):8203–10. doi: 10.1021/acs.energyfuels.5c00396 (PMC12051452; doi:10.1021/acs.energyfuels.5c00396)
Supplement: Supplementary file 1 — ef5c00396_si_001.pdf [file ef5c00396_si_001.pdf]

Supporting Information

Ionomer Interphase Layers Enable Efficient Anion-Exchange Membrane Water Electrolyzer Operation at Low pH

Arthur P. L. Thévenot[1†], Thilo Reiter[1†], Trung Ngo Thanh[1], Lisa Titze[2], Cristina Cazzaniga[2], Fabio Dionigi[1], Peter Strasser[1]

[1]*Technical University of Berlin, Department of Chemistry, Chemical Engineering Division, Str. des 17. Juni 124, 10623 Berlin, Germany*

[2]*Industrie De Nora S.p.A. Via Bistolfi 35, 20134 Milan, Italy*

[†]*AT and TR contributed equally to this work*

Corresponding author:

Prof. Dr. Peter Strasser: pstrasser@tu-berlin

Table of Contents

1 ITL study on anode and both electrodes

Fig. S1: ITL study on anode **3**

Fig. S2: ITL study on both electrodes **4**

2 SEM Characterization

Supplementary Discussion 1 .... **5**

Fig. S3: “as-prepared” electrodes (top view) **6**

Fig. S4: “as-prepared” electrodes (cross-section) **7**

Fig. S5: “post-mortem” benchmark MEA at pH 14 .... **8**

Fig. S6: “post-mortem” benchmark MEA at pH 12 .... **9**

Fig. S7: “post-mortem” ITL MEA at pH 12 .... **10**

Fig. S8: “post-mortem” ICL MEA at pH 12 .... **11**

3 Materials and methods

Fig. S9: Photographs of “as-prepared” electrodes **12**

Fig. S10: Step-by-step assembly of the MEA in the single cell **12**

Fig. S11: Step-by-step connection of the tightened single cell to the electrolyte reservoirs, pumps and potentiostat **13**

Fig. S12: Photographs of the electrolyzer test station **14**

Fig. S13: Activity electrochemical testing protocol **15**

Fig. S14: Stability electrochemical testing protocol **16**

Table S1: Cathodic ionomer content of the studied architectures **17**

4 Conductivity of electrolyte solutions and AEM

Table S2 .... **18**

References **18**

1 ITL study on anode and both electrodes

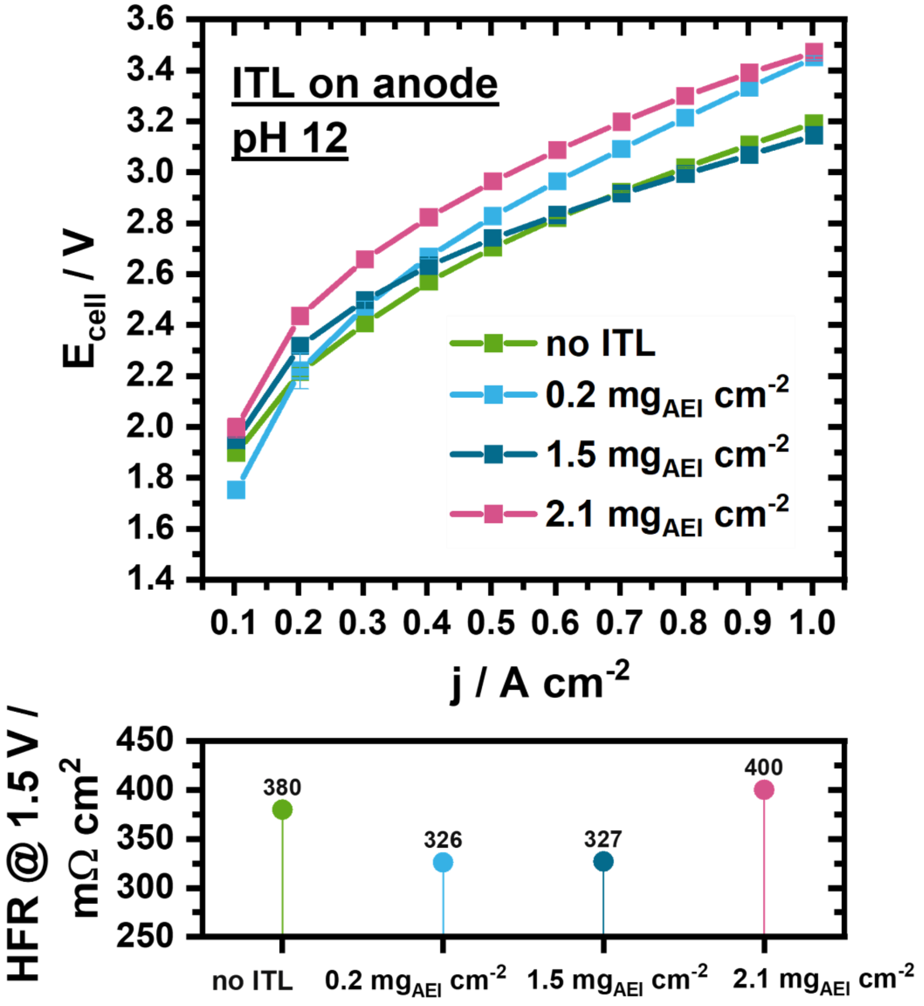

Fig. S1: Study in pH 12 of the ITL applied only over the anode's CL (2.5 mg cm^{-2} NiFe-LDH + 10 wt%$_{total\ solid\ content}$ ionomer) with the following loadings: no ITL (*green*); 0.2 mg$_{AEI}$ cm^{-2} (*cyan*), 1.5 mg$_{AEI}$ cm^{-2} (*dark blue*) and 2.1 mg$_{AEI}$ cm^{-2} (*pink*) ionomer. The respective high-frequency resistance values measured @ 1.5 V are shown below the polarization curves.

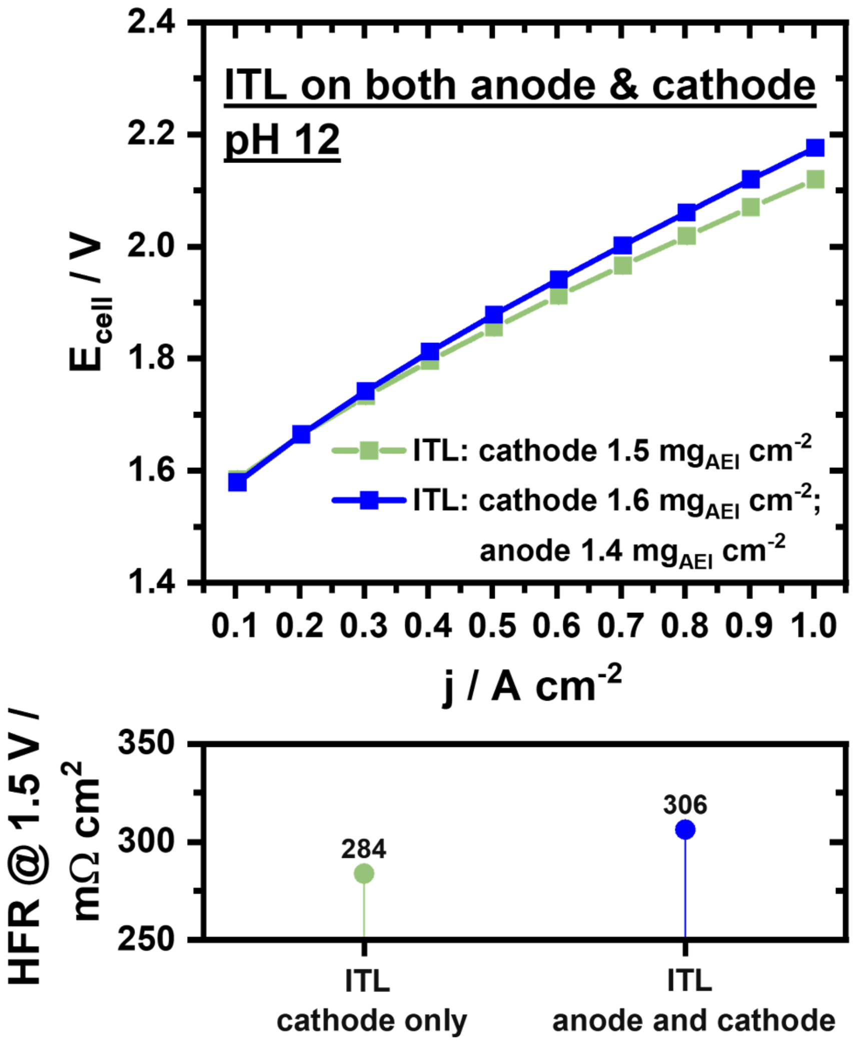

Fig. S2: Performance comparison in pH 12 between using the ITL only over the cathode CL (0.4 mg$_{Pt}$ cm^{-2} Pt/C; ITL loading: 1.5 mg$_{AEI}$ cm^{-2} ionomer; *green*) and over both the cathode CL (ITL loading: 1.5 mg$_{AEI}$ cm^{-2}) and anode CL (2.5 mg cm^{-2} NiFe-LDH + 10 wt%$_{total\ solid\ content}$ ionomer; ITL loading: 1.4 mg$_{AEI}$ cm^{-2}; *blue*). The respective high-frequency resistance values measured @ 1.5 V are shown below the polarization curves.

2 SEM Characterization

Supplementary Discussion 1

The ionomer incorporation affected the catalyst layer morphology of the ICL and ITL cathodes, as shown in **Figure S3** and **Figure S4**. When applied on top of the cathode, following the ITL route, the ionomer does not appear homogeneously distributed on the electrode, but tends to form build-ups on the edge of surface CL plaques. The application of the ionomer has an impact on the flatness of the electrode by opening and lifting up the CL plaques. On cross section, the ionomer is not clearly distinguishable in terms of morphology. When the ionomer is incorporated within the CL, following the ICL approach, the porous aspect of the CL is more pronounced and shows richer aggregates (white spots) which indicate a less homogeneous catalyst distribution compared to the binder-free counterpart.

No significant difference is detectable between the "post-mortem" samples and their "as-prepared" counterparts, regardless the conditions used during the durability measurements. However, the components of the "post-mortem" MEAs tend to separate when the sample is filled with resin for cross-section preparation. Such behavior is particularly observed with the MEA bearing the ICL-cathode (**Fig. S8**): both anode and cathode are separated from the membrane, while a piece of the cathode's CL detached from the PTL and remained on the surface of the membrane. It is therefore difficult to attribute possible surface modifications of the MEA to the single-cell electrochemical measurement itself or the handling in its "post-mortem" form.

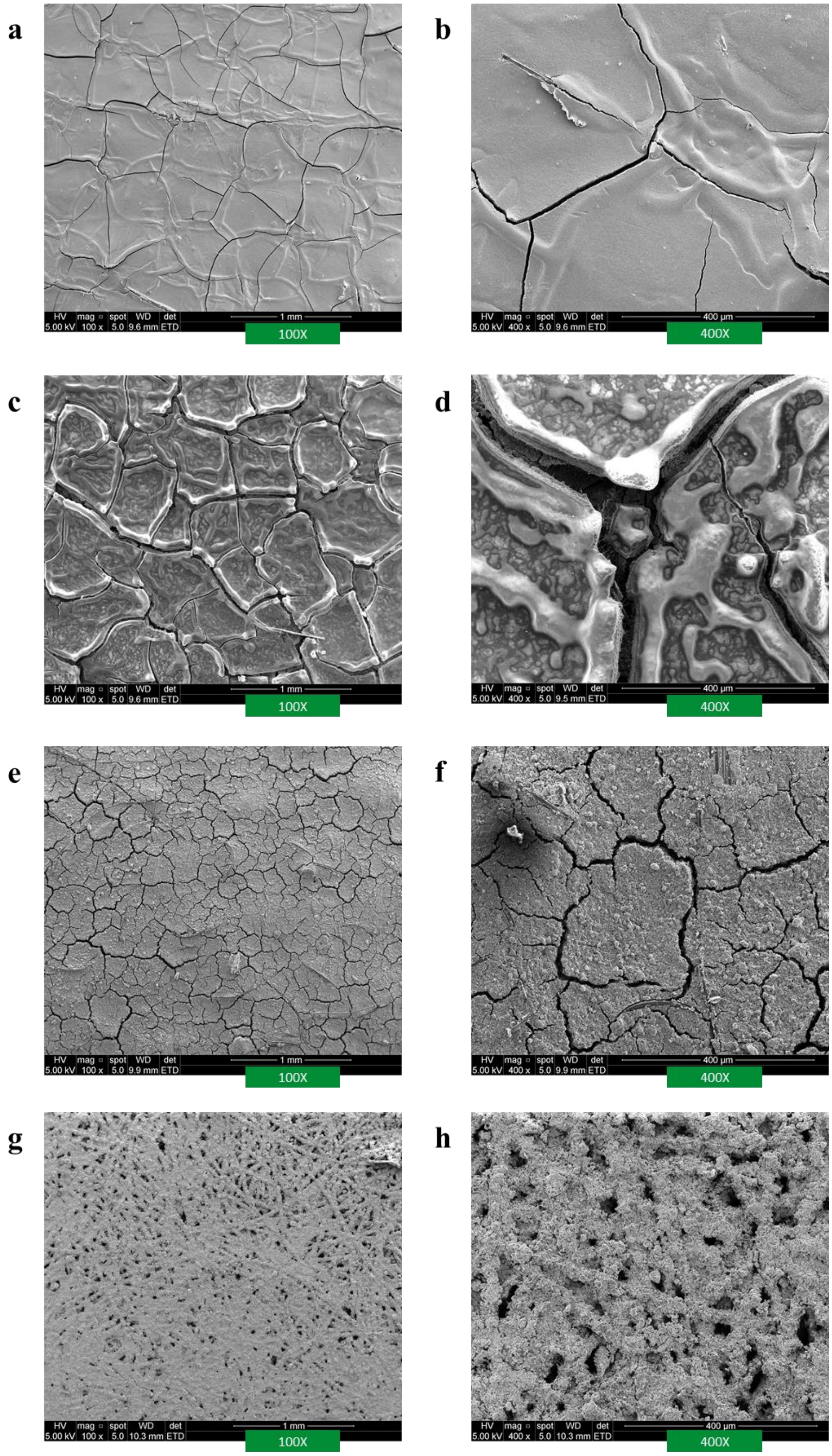

Fig. S3: SEM top-view images of **"as-prepared" electrodes**, at different magnitudes: **(a,b)** ionomer-free cathode; **(c,d)** cathode with ITL (1.5 mg cm^{-2} ionomer); **(e,f)** cathode with ICL (0.08 mg cm^{-2} ionomer = 20 wt% with respect to Pt); **(g,h)** anode with ICL (2.5 mg cm^{-2} NiFe-LDH + 10 $wt\%_{\text{total solid content}}$ ionomer).

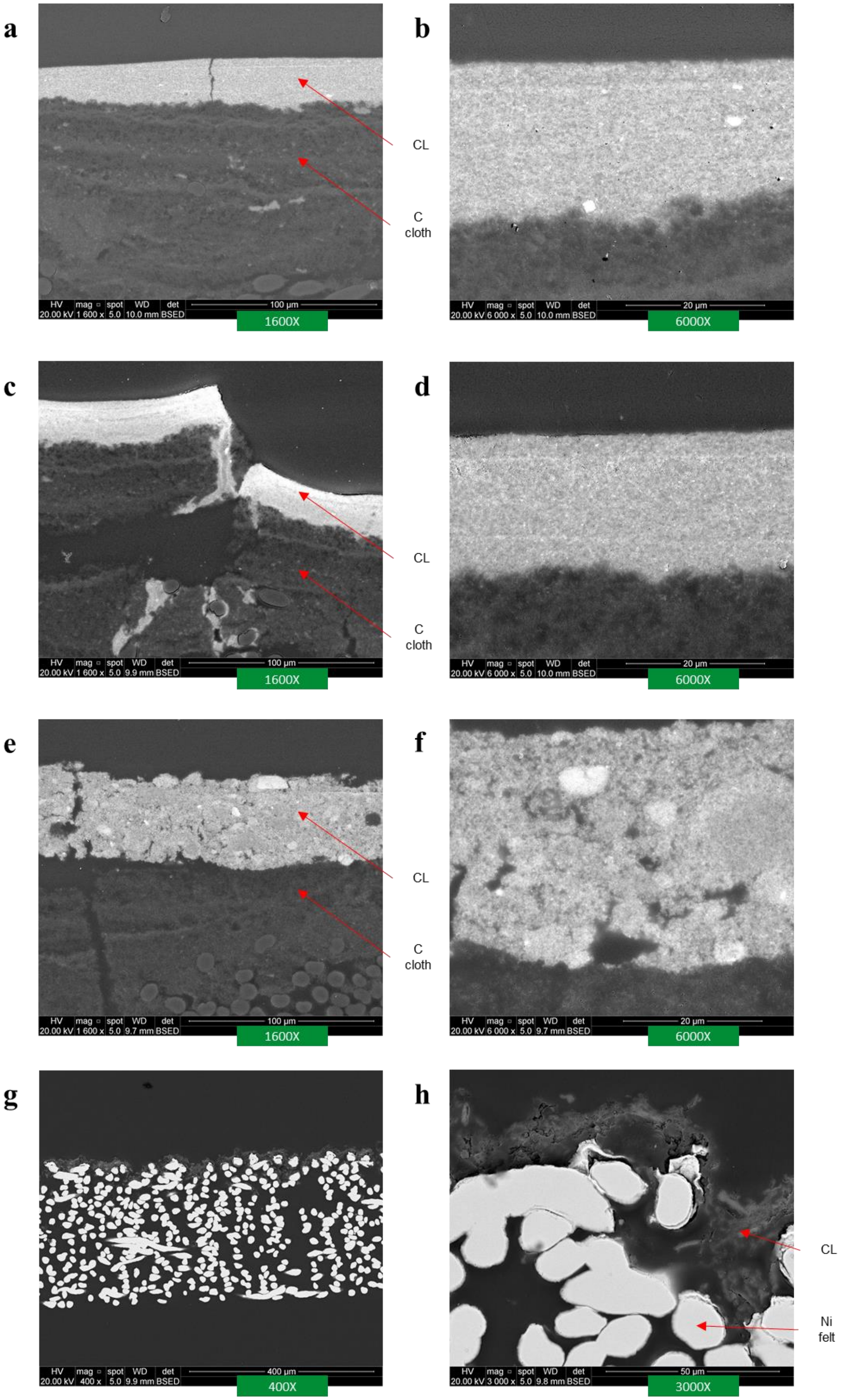

Fig. S4: SEM cross-section images of **"as-prepared" electrodes**, at different magnitudes: **(a,b)** ionomer-free cathode; **(c,d)** cathode with ITL (1.5 mg cm^{-2} ionomer); **(e,f)** cathode with ICL (0.08 mg cm^{-2} ionomer = 20 wt% with respect to Pt); **(g,h)** anode with ICL (2.5 mg cm^{-2} NiFe-LDH + 10 wt%$_{\text{total solid content}}$ ionomer.

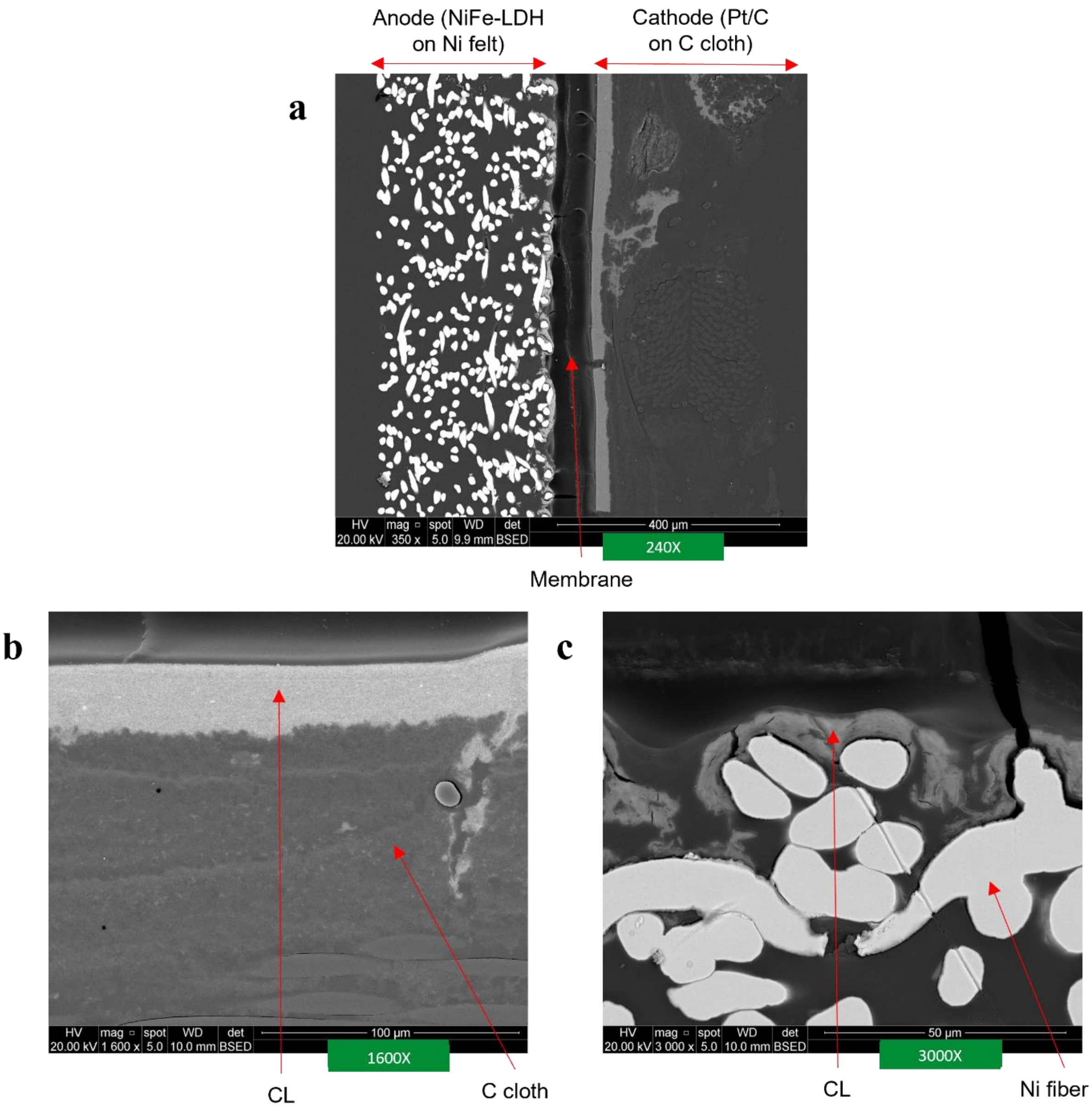

Fig. S5: SEM cross-section images of **"post-mortem" benchmark MEA after 1 M KOH (pH 14)** durability test: **(a)** MEA; **(b)** ionomer-free cathode; **(c)** anode.

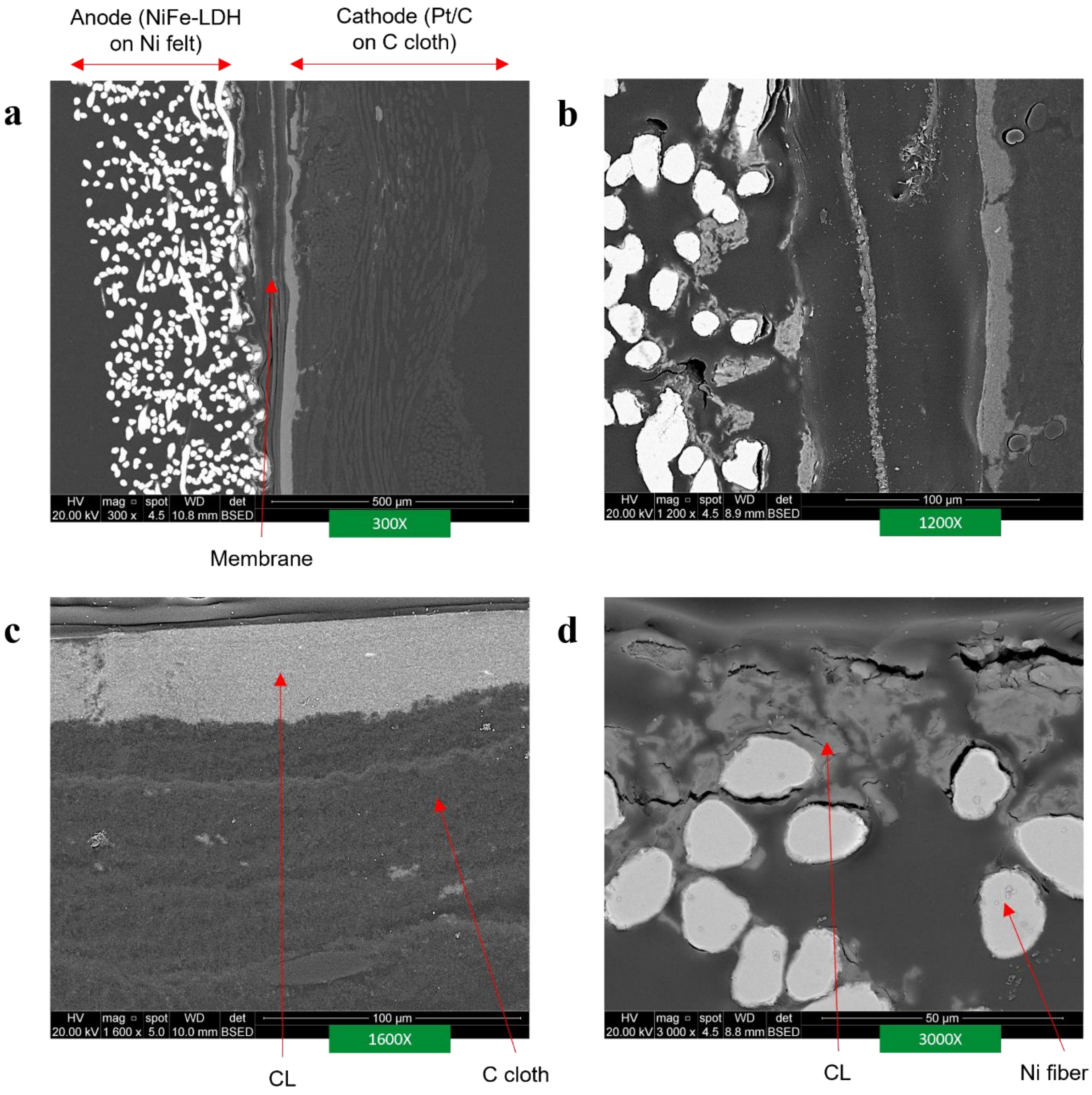

Fig. S6: SEM cross-section images of **"post-mortem" benchmark MEA after <u>0.01 M KOH (pH 12)</u>** durability test: **(a)** MEA; **(b)** MEA closed-up view; **(c)** ionomer-free cathode (CL thickness average = 26.10 ± 5.30 μm); **(d)** anode (CL thickness average = 15.10 ± 6.50 μm).

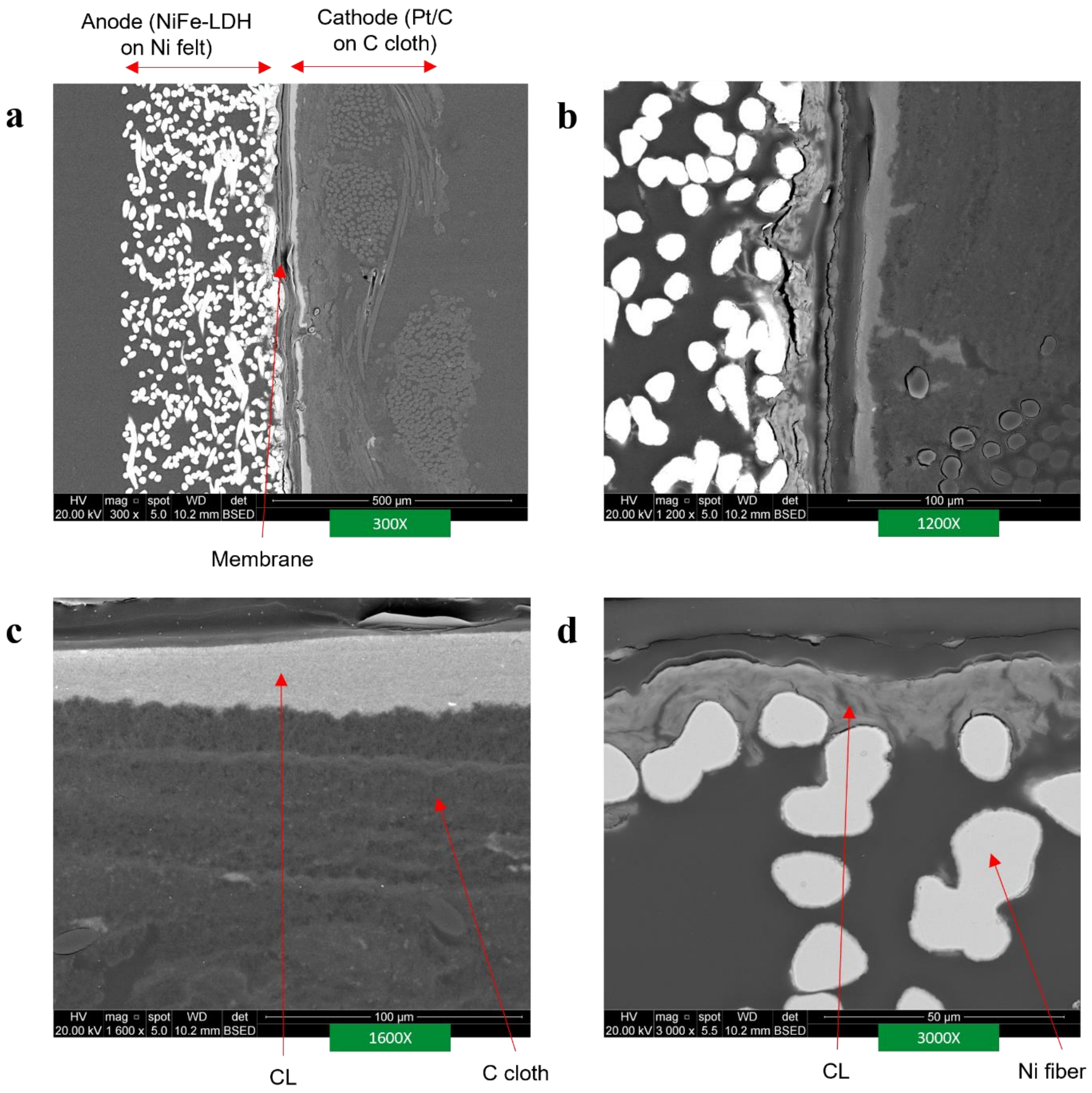

Fig. S7: SEM cross-section images of **"post-mortem" MEA with <u>ITL-cathode (1.5 mg cm^{-2} ionomer)</u>** durability test in **<u>0.01 M KOH (pH 12)</u>**: **(a)** MEA; **(b)** MEA closed-up view; **(c)** cathode with ITL (CL+ITL thickness average = 27.10 ± 4.90 μm); **(d)** anode (CL thickness average = 13.20 ± 6.20 μm).

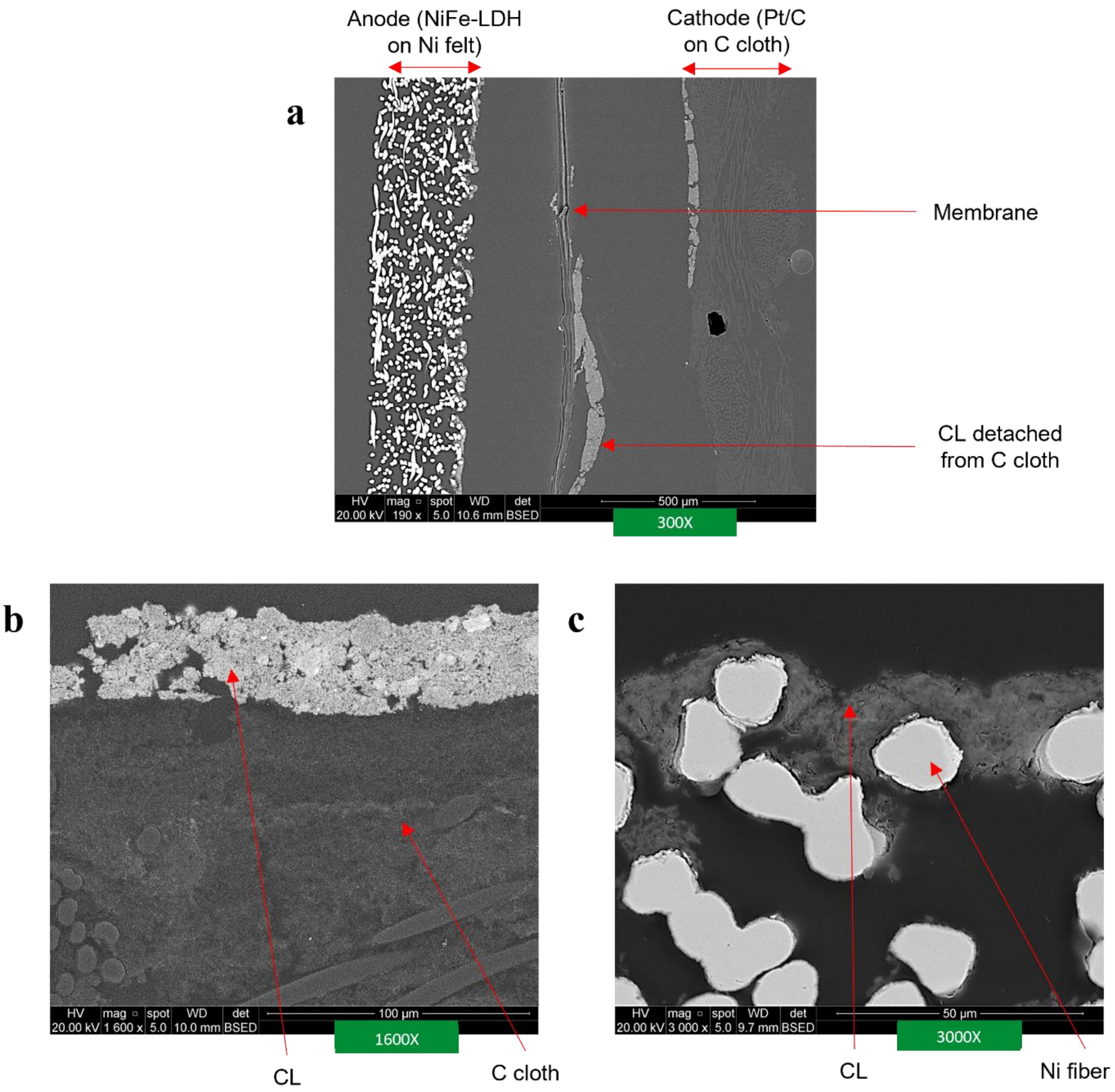

Fig. S8: SEM cross-section images of **"post-mortem" MEA with <u>ICL-cathode (0.08 mg cm^{-2} ionomer = 20 wt % with respect to Pt)</u>** durability test in **<u>0.01 M KOH (pH 12)</u>**: **(a)** MEA; **(b)** MEA closed-up view; **(c)** cathode with ICL (CL thickness average = 33.40 ± 5.50 µm); **(d)** anode (CL thickness average = 18.9 ± 11.80 µm).

3 Materials and methods

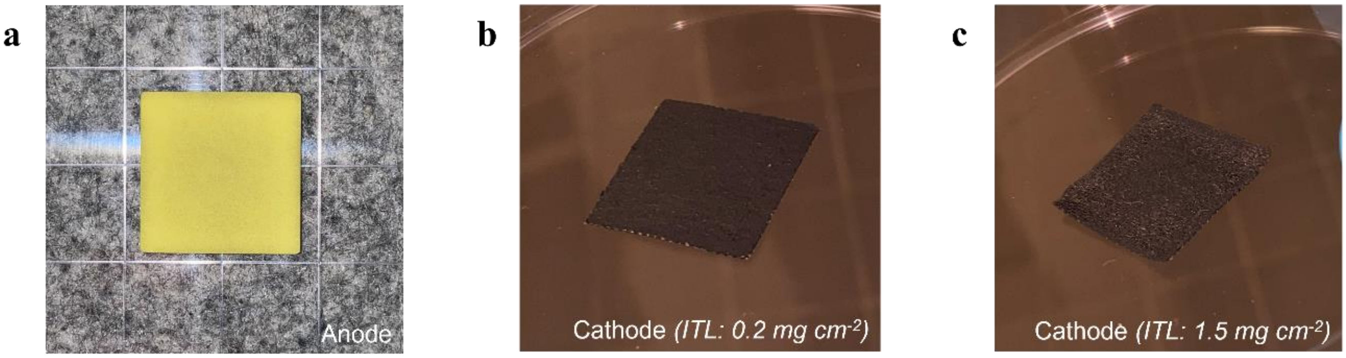

Fig. S9: Photographs of "as-prepared" PTLs: **(a)** anode with CL; binder-free cathodes with **(b)** 0.2 mg_{AEI} cm^{-2} and **(c)** 1.5 mg_{AEI} cm^{-2} ITL.

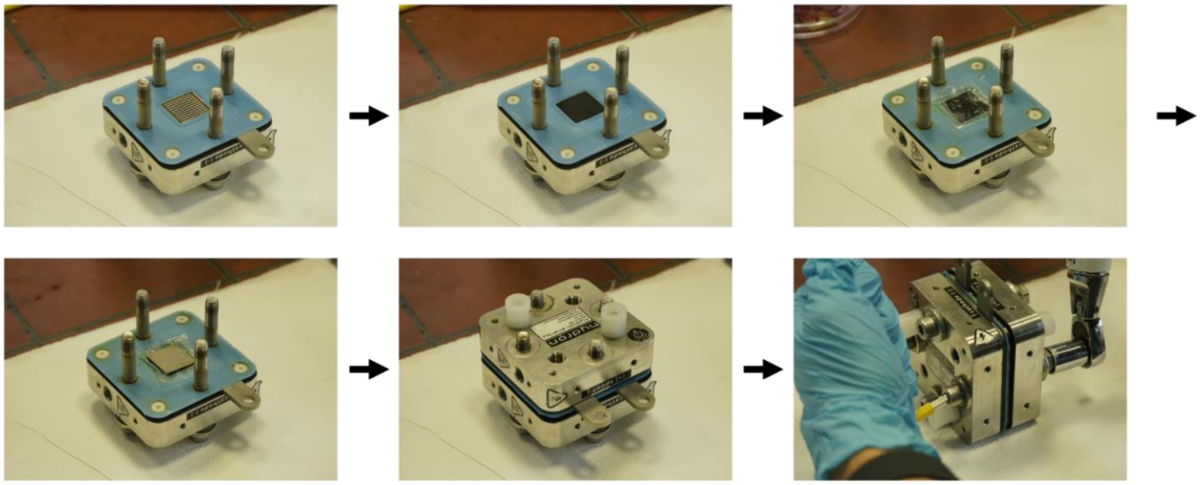

Fig. S10: Step-by-step assembly of the MEA in the single cell.

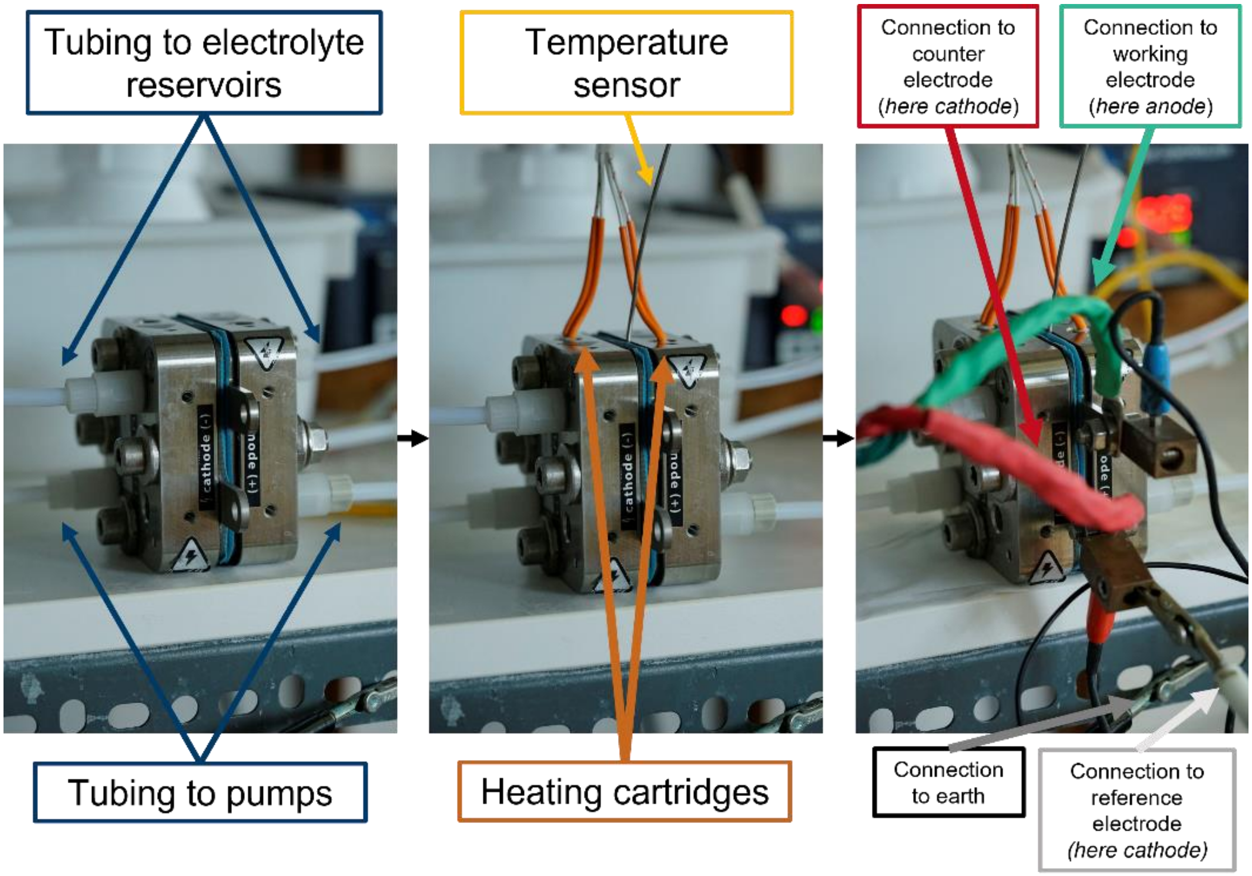

Fig. S11: Step-by-step connection of the tightened single cell to the electrolyte reservoirs, pumps and potentiostat.

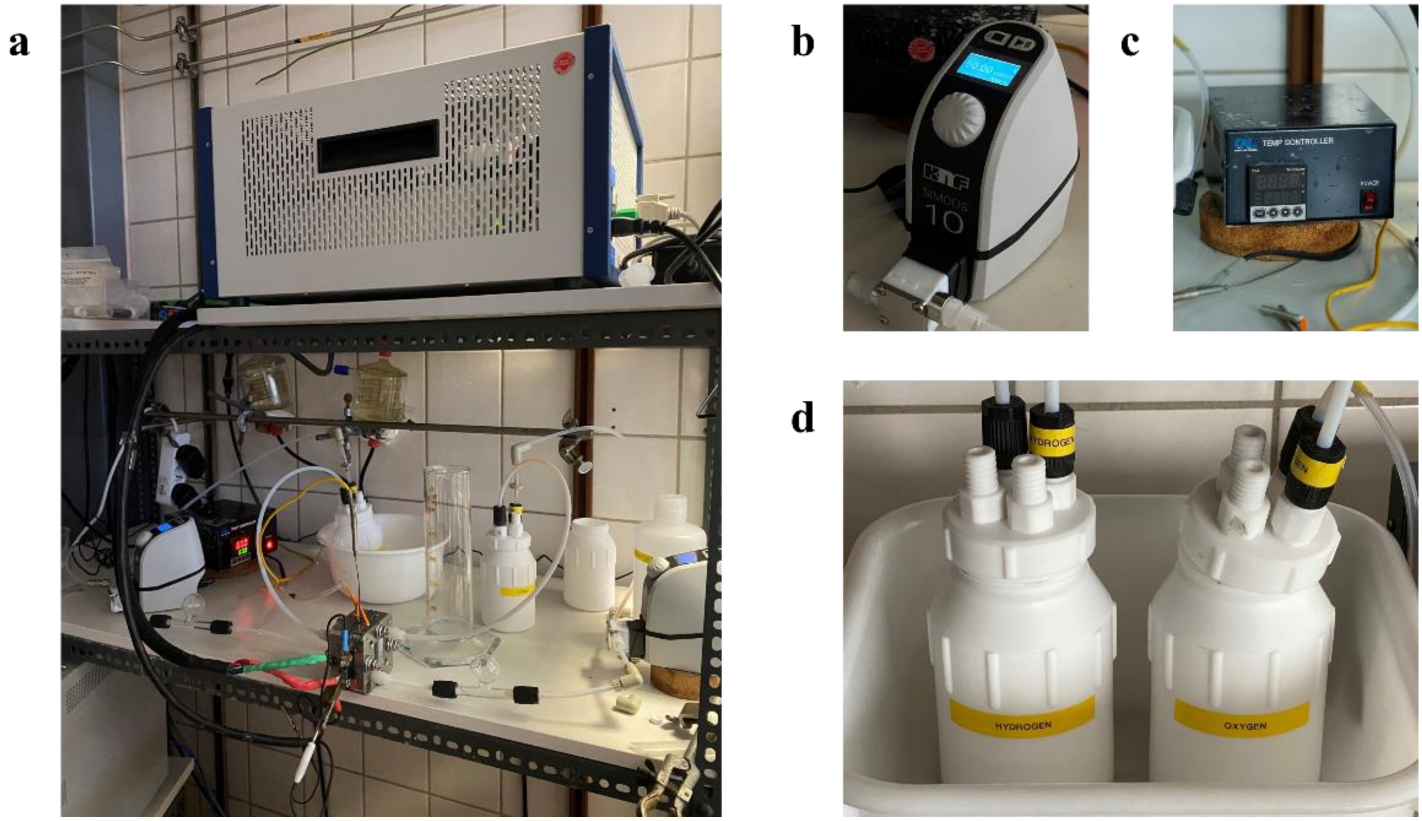

Fig. S12: Photographs of the electrolyzer test station: **(a)** overview of the test station during a durability test, showing potentiostat, electrolyte reservoirs, pumps and single cell; **(b)** pump (anode side) under operation; **(c)** temperature controller (shown switched off); **(d)** electrolyte reservoirs (caps are tightened during operation).

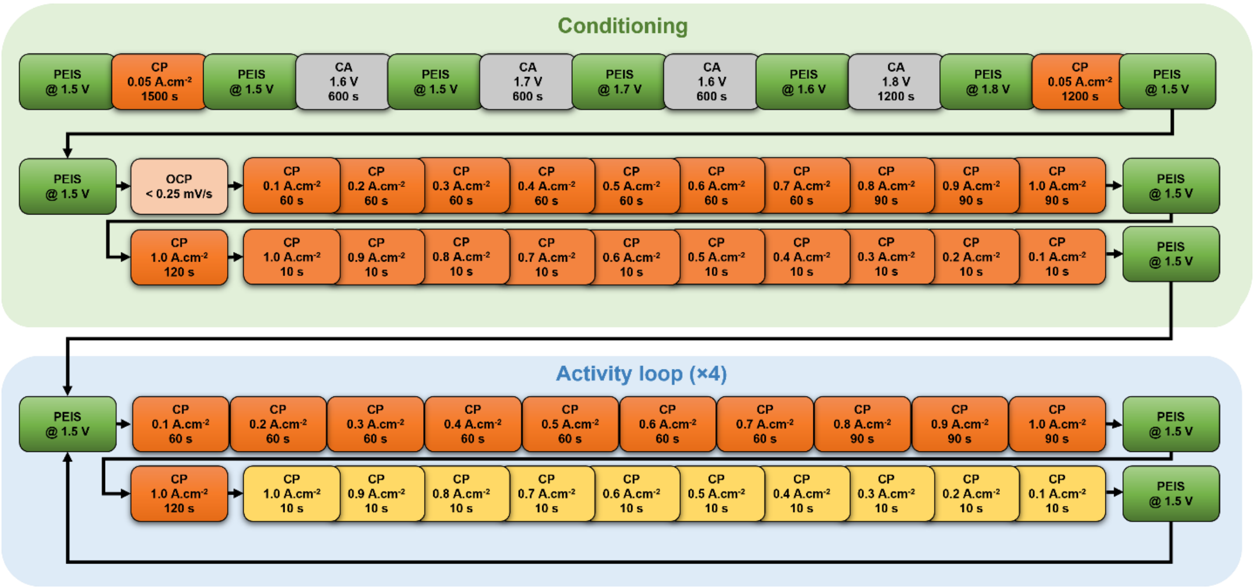

Fig. S13: Activity electrochemical testing protocol. Each reported CP value point was obtained from averaging the corresponding 10 s CP step (*yellow box*) of three consecutive loops. HFR values were evaluated from EIS measurements by the determination of the x-axis intercept of the high frequency data (Nyquist plot). Each reported value was obtained from averaging the HFRs from the three PEIS steps (*green boxes*) of three consecutive loops (*blue section*).

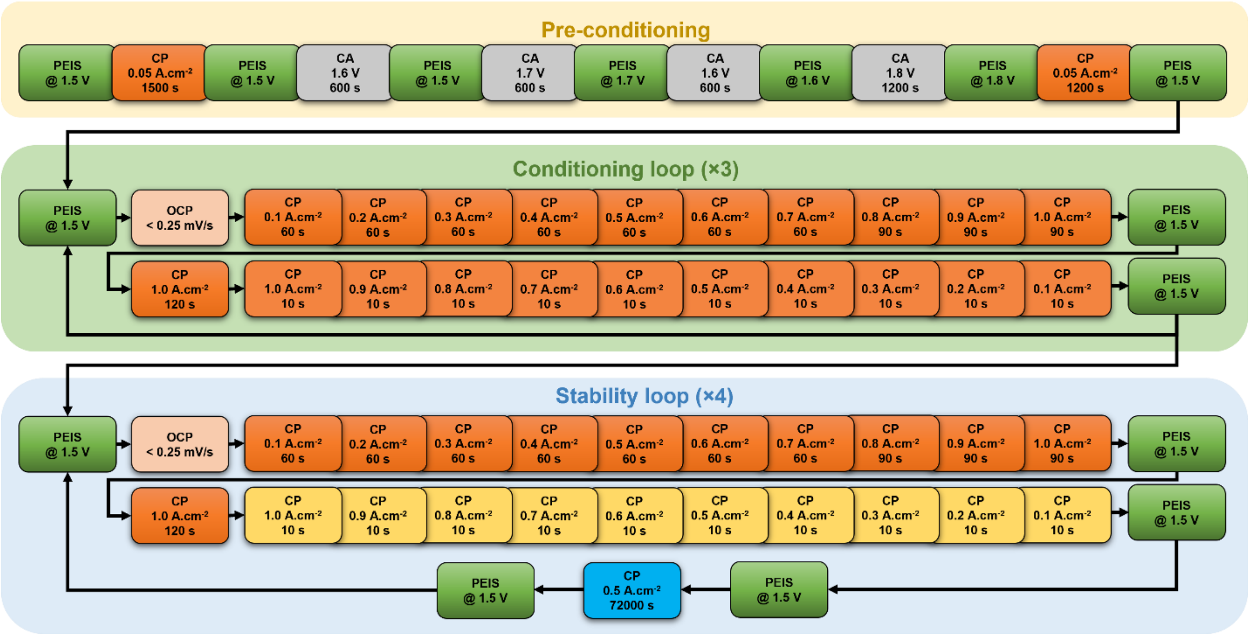

Fig. S14: Stability electrochemical testing protocol. The reported 20 h-CPs (*cyan box*) are performed at the end of each "Stability loop" (*blue section*). HFR values were evaluated analogously to the activity protocol. Each reported HFR value was obtained from the last PEIS (*green box following the 20 h-CP step*) of each "Stability loop".

Table S1: Cathodic ionomer content of the studied architectures, using the following metrics: ionomer geometric mass (mg_{AEI} cm^{-2}); ionomer weight percentage with respect to platinum content (wt%); ionomer-to-catalyst ratio with respect to platinum content (I/C).

Cathodic Ionomer Architecture	Geometric Mass (mg_{AEI} cm^{-2})	Weight Percentage (wt%)	Ionomer/Catalyst Ratio (I/C)
ICL	0.02	5	0.05
	0.04	10	0.1
	0.08	20	0.2
ITL	0.08	20	0.2
	0.3	75	0.75
	1.5	375	3.75
	4.0	1000	10

4 Conductivity of electrolyte solutions and AEM

Table S2: Conductivity (mS cm^{-2}) at 25 °C and 60 °C of 1 M KOH, 0.1 M KOH, 0.01 M KOH electrolyte solutions and Sustainion® X37 membrane after ion exchange in 1 M KOH solution.[1-3]

AEM / Electrolyte	Conductivity (mS cm^{-1}) @ 25 °C	Conductivity (mS cm^{-1}) @ 60 °C	References
1 M KOH	215.3	322.2	Calculated values from R.J. Gilliam *et al.*[1]
0.1 M KOH	24.1	35.4	
0.01 M KOH	2.4	3.6	
Sustainion® X37 membrane	64 *(in 1 M KOH)*[2]	116 *(in 1 M KOH)*[3]	Liu *et al.*[2] Dioxide Materials[3]

References:

(1) Gilliam, R. J.; Graydon, J. W.; Kirk, D. W.; Thorpe, S. J. A review of specific conductivities of potassium hydroxide solutions for various concentrations and temperatures. *International Journal of Hydrogen Energy* **2007**, *32* (3), 359-364. DOI: https://doi.org/10.1016/j.ijhydene.2006.10.062.
(2) Liu, Z.; Yang, H.; Kutz, R.; Masel, R. I. CO2Electrolysis to CO and O2at High Selectivity, Stability and Efficiency Using Sustainion Membranes. *Journal of The Electrochemical Society* **2018**, *165* (15), J3371-J3377. DOI: 10.1149/2.0501815jes.
(3) Dioxide Materials. https://dioxidematerials.com/products/anion-exchange-membranes/.
